# Machine learning‐based identification of a cell death‐related signature associated with prognosis and immune infiltration in glioma

**DOI:** 10.1111/jcmm.18463

**Published:** 2024-06-07

**Authors:** Quanwei Zhou, Fei Wu, Wenlong Zhang, Youwei Guo, Xingjun Jiang, Xuejun Yan, Yiquan Ke

**Affiliations:** ^1^ The National Key Clinical Specialty, Department of Neurosurgery Zhujiang Hospital, Southern Medical University Guangzhou China; ^2^ Department of Neurosurgery Xiangya Hospital, Central South University Changsha China; ^3^ NHC Key Laboratory of Birth Defect for Research and Prevention Hunan Provincial Maternal and Child Health Care Hospital Changsha Hunan China

**Keywords:** cell death, glioma, immune infiltration, machine learning, prognosis, tumour microenvironment

## Abstract

Accumulating evidence suggests that a wide variety of cell deaths are deeply involved in cancer immunity. However, their roles in glioma have not been explored. We employed a logistic regression model with the shrinkage regularization operator (LASSO) Cox combined with seven machine learning algorithms to analyse the patterns of cell death (including cuproptosis, ferroptosis, pyroptosis, apoptosis and necrosis) in The Cancer Genome Atlas (TCGA) cohort. The performance of the nomogram was assessed through the use of receiver operating characteristic (ROC) curves and calibration curves. Cell‐type identification was estimated by using the cell‐type identification by estimating relative subsets of known RNA transcripts (CIBERSORT) and single sample gene set enrichment analysis methods. Hub genes associated with the prognostic model were screened through machine learning techniques. The expression pattern and clinical significance of MYD88 were investigated via immunohistochemistry (IHC). The cell death score represents an independent prognostic factor for poor outcomes in glioma patients and has a distinctly superior accuracy to that of 10 published signatures. The nomogram performed well in predicting outcomes according to time‐dependent ROC and calibration plots. In addition, a high‐risk score was significantly related to high expression of immune checkpoint molecules and dense infiltration of protumor cells, these findings were associated with a cell death‐based prognostic model. Upregulated MYD88 expression was associated with malignant phenotypes and undesirable prognoses according to the IHC. Furthermore, high MYD88 expression was associated with poor clinical outcomes and was positively related to CD163, PD‐L1 and vimentin expression in the in‐horse cohort. The cell death score provides a precise stratification and immune status for glioma. MYD88 was found to be an outstanding representative that might play an important role in glioma.

## 1 INTRODUCTION

Glioma is the most common primary intracranial nerve tumour. Moreover, glioma is a heterogeneous disease entity that may include multiple clinically and biologically distinct subtypes. However, glioma patients with similar molecular and histological characteristics may have distinct clinical prognoses and treatment responses, which indicates that additional factors may be involved.^1^, ^2^ In the past 20 years, despite aggressive therapy, including surgical resection and chemoradiotherapy, gliomas, especially glioblastoma, are highly lethal, with a median survival time of less than 2 years.^3^ Although recent studies have demonstrated that immune checkpoint inhibitors are highly effective at treating other tumours,^4^ only a minority of patients are likely to benefit from current immunotherapy,^5^ due in part to an inability to elicit inflammatory cell death.^6^ The immunosuppressive environment of glioma limits the effectiveness of immunotherapy, and additional and combination approaches are likely required for continued beneficial effects.^7^ The existing treatment regimens are hard to further improve the clinical outcomes of glioma patients and novel therapeutic targets are needed to improve the prognosis of those patients. Thus, it is urgently required to establish a new prognostic evaluation model to predict the prognosis of glioma and Identification of glioma populations and molecular targets suitable for immunotherapy.

Recently, it has been reported that different cell death patterns, such as cuproptosis, ferroptosis, pyroptosis, apoptosis and necrosis, are closely related to the tumour immune and play an important role in tumour progression, which are promising for predicting tumour prognosis.^8^ Dying or dead cells can produce various ‘find‐me/eat‐me’ signals for immune cells to locate, migrate and clear away.^9^ The types of cell death were classified into accidental cell death (necrosis) and regulated cell death (programmed cell death, PCD).^10^ Inducing ferroptosis has shown to be feasible in glioma treatment.^11^ Calreticulin (CRT), a ferroptosis‐related protein, was reported to be involved in regulating the tumour microenvironment.^12^ Ferroptosis favours and facilitates the translocation of CRT to tumour cell surfaces, where CRT can function as an ‘eat‐me’ signal and activate powerful antitumor responses.^13^ Cuproptosis is a newly identified type of PCD induced by copper.^14^ However, the role of cuproptosis in glioma development and its microenvironment is not fully understood. Apoptosis is among the best‐characterized types of PCD,^15^ however, various other nonapoptotic types of PCD may occur during the progression of cancer. Anticancer therapies that induce inflammatory cell death to activate immunity are known to be effective. The importance of immunogenic apoptosis deserves further exploration. Pyroptosis and necroptosis have emerged as two additional forms of immunogenic apoptosis. Pyroptosis is induced by inflammasomes and mediated by masterminds. It is characterized by modulating the clearance of pathogens from infections and is caspase‐dependent.^16^ Accumulating evidence has elucidated the fundamental role of necroptosis in tumorigenesis and metastasis and suggested its potential as a novel anticancer target.^17^ However, the specific roles of different cell death in glioma have been fewer studies. Therefore, a better understanding of different cell death pattern‐related genes helps investigate potential biomarkers and paves the way for developing new strategies for glioma immunotherapy.

In this study, a scoring system was developed and validated in six cohorts by a machine learning‐based integrative procedure, in addition, we compared our model with other published models and showed the superiority of this model, furthermore, we identified that MYD88 was a hub gene associated with a cell death‐based prognostic model and the expression of MYD88 was closely associated with the expression of Immune checkpoint key molecule, such as vimentin, HIF1A, CD163, PD‐L1, CD40 and STAT3, which provides a precise stratification and predicts the efficacy of immunotherapy for glioma.

## 2 METHODS

### 2.1 The acquisition and processing of data

We extracted transcriptome data and relevant clinical information from six public datasets, namely, the Cancer Genome Atlas (TCGA), GSE16011, CCGA‐693, CCGA‐301, CCGA‐325 and Rembrandt datasets. The data processing and acquisition were performed according to previous methods in the literature,^18^ and the flowchart of data collection and method implementation is shown in Figure S3.

### 2.2 Human specimens

A total of 177 glioma tissues, 30 tumour tissues, paired paratumour tissues and 17 normal brain tissues from patients with traumatic brain injury were harvested from Xiangya Hospital, Central South University. All participants provided written informed consent, and ethics approval was obtained from the ethics committee of Xiangya Hospital, Central South University. The pertinent data are shown in Table 1.

**TABLE 1:** Clinical characteristics of the in‐house cohort.

Characteristics	In‐house cohort (*n* = 177)
Age	
Mean ± SD	44.91 ± 14.12
Median [min–max]	47.00 [18.00,75.00]
Sex, *n* (%)	
Female	73 (41.24%)
Male	104 (58.76%)
WHO grade, *n* (%)	
2	43 (24.29%)
3	38 (21.47%)
4	83 (46.89%)
na	13 (7.34%)
IDH status, *n* (%)	
Mutant	59 (33.52%)
Wild‐type	99 (56.25%)
na	18 (10.23%)
Histology, *n* (%)	
Astrocytoma	84 (47.46%)
GBM	81 (45.76%)
Oligodendroglioma	12 (6.78%)
Radiotherapy, *n* (%)	
No	50 (28.41%)
Yes	104 (59.09%)
na	22 (12.50%)
Chemotherapy, *n* (%)	
No	48 (27.27%)
Yes	106 (60.23%)
na	22 (12.50%)
Laterality, *n* (%)	
Both	5 (2.82%)
Left	76 (42.94%)
Middle	9 (5.08%)
Right	87 (49.15%)
Tumour location, *n* (%)	
Brainstem	3 (1.69%)
Cerebellar	6 (3.39%)
Frontal	86 (48.59%)
Insular	5 (2.82%)
Occipital	9 (5.08%)
Parietal	16 (9.04%)
Sellar	4 (2.26%)
Temporal	45 (25.42%)
Thalamus	3 (1.69%)

### 2.3 Immunohistochemistry (IHC)

A tissue microarray was constructed with 78 glioma tissues. IHC was carried out using primary antibodies against HIF1A (CST, USA), STAT3 (Proteintech, China), VIM (Proteintech, China), CD40 (Proteintech, China), PD‐L1 (CST, USA), CD163 (Proteintech, China) and MYD88 (Proteintech, China). The IHC analysis was performed as described previously.^18^

### 2.4 Identification of cell death‐related genes significantly associated with prognosis

The cell death‐related gene list was obtained from previous studies in the literature^13^, ^19^, ^20^ (Table S1). A total of 534 genes were subjected to Kaplan–Meier survival analysis in the six public datasets, and 139 overlapping genes were significantly associated with prognosis, with a cutoff criterion of *p* < 0.05 (Table S2).

### 2.5 Artificial intelligence‐derived prognostic signature

We randomly divided the TCGA dataset at a ratio of 7:3 for the training and validation sets. For the training cohort, a scoring system was established by using the logistic regression model with the shrinkage regularization operator (LASSO) Cox regression model to identify 21 prognostic markers via R package ‘glmnet’ among 139 genes in the training cohorts,^21^, ^22^ which were verified in the other cohorts. We removed eight genes after performing a pairwise correlation analysis of 21 genes. The remaining 13 genes were screened using machine learning. We then integrated seven classical algorithms^23^: random forest (RSF), gradient boosting machine (GBM), survival support vector machine (Survival‐SVM), supervised principal component (SuperPC), partial least squares regression for Cox (plsRcox), CoxBoost and stepwise Cox. We used public cohorts with larger sample sizes as the training cohort and used seven algorithms to construct signatures separately in the expression files with 12 prognostic markers. Finally, in the CGGA‐301, CGGA‐325, GSE16011, CGGA and Rembrandt cohorts, we calculated the risk score for each cohort using the signature obtained from the TCGA cohort. Based on the average C‐index of the six cohorts, we ultimately selected the best consensus prognostic model for glioma. We also compared the performance of the artificial intelligence‐derived prognostic signature with that of 10 published signatures in predicting patient prognosis.^24^, ^25^, ^26^, ^27^, ^28^, ^29^, ^30^, ^31^, ^32^, ^33^

### 2.6 Validation and evaluation of the scoring system

The individual risk score was estimated based on the coefficient and mRNA expression. The median risk score served as a threshold for dichotomizing patients into high‐ and low‐risk prognostic groups. The R packages ‘survminer’ and ‘survival’ were used to generate Kaplan–Meier curves to investigate differences in survival between the prognostic groups. A nomogram integrating clinical variables was created with the ‘rms’ package in R software. The predictive accuracy of the models was subsequently assessed by receiver operating characteristic (ROC) curve analysis and calibration curve analysis via the ‘survivalROC’ and ‘rms’ packages.

### 2.7 Differential expression analyses and functional enrichment analysis

Based on the differential expression of genes (DEGs) (|log2FC| ≥ 1, *q* value <0.05) between the two groups, Gene Ontology (GO) and Kyoto Encyclopaedia of Genes and Genomes (KEGG) functional enrichment analyses were performed with the R package ‘clusterProfiler’. Gene set enrichment analysis (GSEA) was performed to investigate the underlying biological processes of the high‐risk and low‐risk groups with GSEA software (version 3.0), which was downloaded from the Broad Institute (http://www.broadinstitute.org/gsea).

### 2.8 Immune and stromal cell infiltration analysis

The cell‐type identification by estimating relative subsets of known RNA transcripts (CIBERSORT) algorithm was applied to deconvolute the bulk RNA‐seq profiles into 22 cell‐type scores for each glioma sample.^34^ The abundances of protumorigenic immune cell subsets (CD56dim NK cells, immature DCs, MDSCs, neutrophils, plasmacytoid DCs, Tregs, Th2 cells and M2 macrophages) were assessed by using the single sample gene set enrichment analysis method for constructing the cell landscape of glioma in the four datasets.^2^, ^35^

### 2.9 Identification of hub genes by machine learning

The root mean squared error is a measure of how well the machine learns the model and was calculated by taking the square root of the average of the residuals (errors not explained by the regression equation) over the total sample size.

### 2.10 Statistical analysis

Two‐group comparisons were analysed using paired or unpaired Student's *t*‐tests. Spearman correlation analysis was performed to determine significant differences and correlations. All statistical analyses were performed with R software (www.r‐project.org). *p* < 0.05, *p* < 0.01, *p* < 0.001 and *p* < 0.0001 were considered to indicate statistical significance.

## 3 RESULTS

### 3.1 Construction and validation of the cell death‐related risk signature for glioma

**FIGURE 1:**
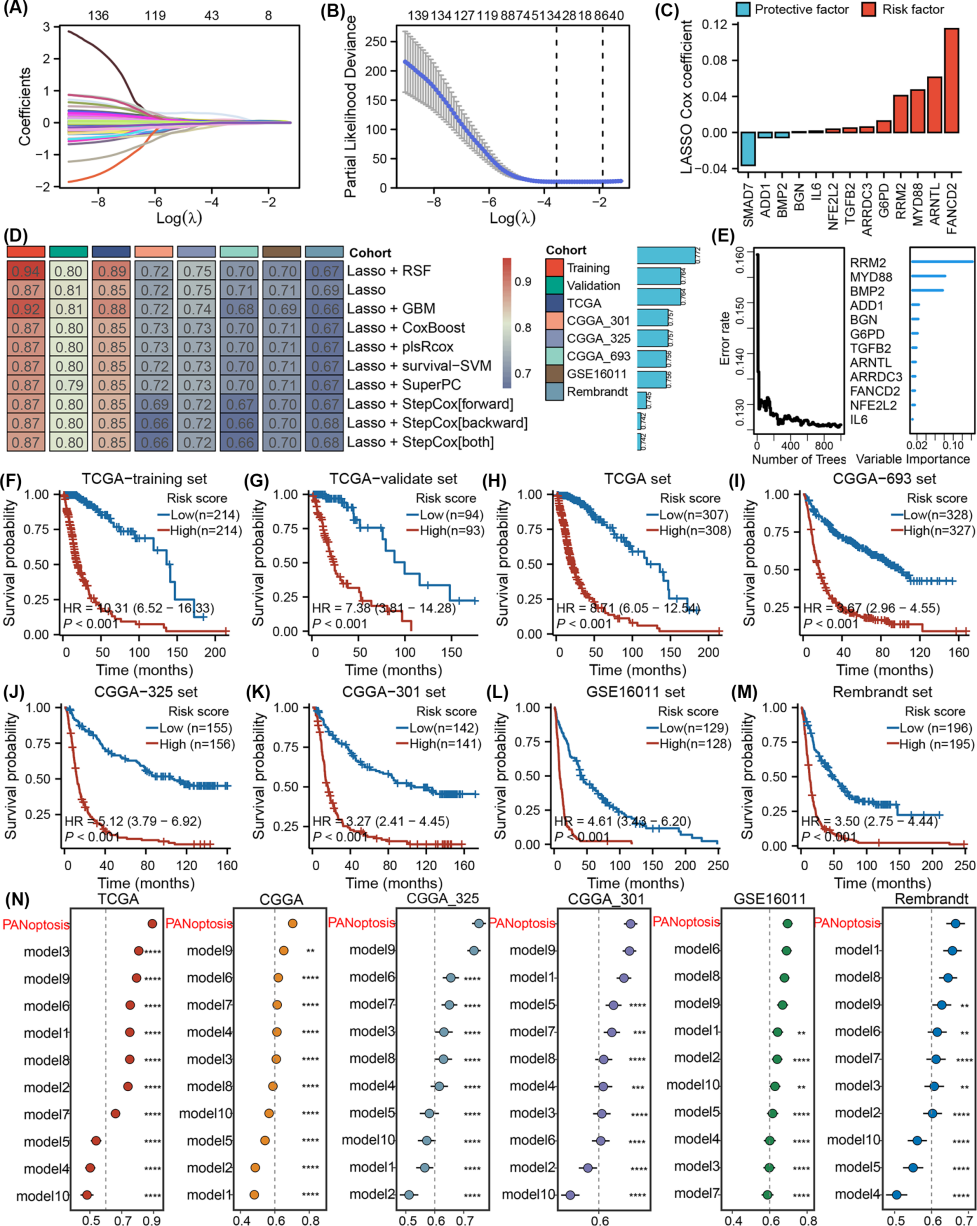
Identification of the cell death‐associated signature in the Cancer Genome Atlas (TCGA) cohort (A) The partial likelihood deviation distribution of the logistic regression model with the shrinkage regularization operator (LASSO) coefficient; (B) partial likelihood deviance revealed by the LASSO regression model; (C) an ensemble of 13 cell death‐associated genes with Cox coefficients; (D) a total of eight machine learning algorithms for the cell death‐associated signatures via a 10‐fold cross‐validation framework. The C‐index of each model was calculated across the validation datasets. (E) The number of trees for determining the cell death‐associated signature with minimal error and the importance of the eight most valuable cell death‐associated signatures based on the RSF algorithm. (F–M) Kaplan–Meier survival curves of OS between high‐risk and low‐risk patients identified by a cell death‐related scoring system in the TCGA training (F), TCGA validation (G), TCGA (H), CGGA‐693 (I), CGGA‐325 (J), CGGA‐301 (K), GSE16011 (L) and Rembrandt (M) cohorts. (N) The C‐index of the cell death‐associated signature and other models developed in the six cohorts.

A graphic abstract of this study is presented in Figure S1. We used survival curves to analyse the relationships between 549 cell death‐associated genes and outcomes in six available public glioma cohorts (CGGA, CGGA‐301, CGGA‐325, TCGA, GSE16011 and the Rembrandt cohort). Kaplan–Meier analysis identified 434 genes that were significantly associated with prognosis in the TCGA cohort, 339 genes in the CGGA cohort, 295 genes in the GSE16011 cohort, 345 genes in the Rembrandt cohort, 317 genes in the CGGA‐301 cohort, and 388 genes in the CGGA‐325 cohort (Table S1). Ultimately, 139 shared genes were identified in the six glioma cohorts (Table S2). Next, a LASSO Cox regression model was applied to screen 21 useful factors for developing a cell death‐related classifier in the TCGA training set (Figure 1A,B, Table S3). Moreover, pairwise correlation analysis revealed similar expression patterns among the genes (Table S4). Ultimately, CAPG, SOCS1, CASP6, CASP2, TMBIM1, NCF2, TIMP1 and STEAP3 were removed, and the remaining 13 genes were used for model construction. Then, seven machine learning algorithms, namely, RSF, stepwise Cox, CoxBoost, plsRcox, SuperPC, GBM and survival‐SVM, were combined based on tenfold cross‐validation to identify the most robust signature with the highest C‐index in the six datasets (Figure 1D,E). We established the best performance for the final 12 cell death‐related genes by combining the RSF and LASSO algorithms. Kaplan–Meier analysis of the training cohort indicated that patients with low scores had significantly better outcomes than those with high scores (HR = 10.31, *p* < 0.001; Figure 1F). Similar findings were detected in the other cohorts (validate set, HR = 7.83; TCGA, HR = 8.71; CGGA‐695, HR = 3.67; CGGA‐325, HR = 5.12; CGGA‐301, HR = 3.27; GSE16011, HR = 4.61; Rembrandt, HR = 3.50) (*p* < 0.001; Figure 1G–M). Therefore, a high cell death score was correlated with a poor prognosis. The area under the curve (ROC) values for 1 year, 3 years and 5 years were 0.899, 0.914 and 0.883, respectively, in the TCGA cohort, which indicated that the prognostic model was highly effective at distinguishing favourable and unfavourable prognoses in glioma patients. Importantly, this cell death‐related signature achieved similar and stable predictive accuracy in the independent cohort (CGGA‐693 cohort, AUC at 1 year: 0.681; at 3 years: 0.736; at 5 years: 0.742; CGGA‐301 cohort, AUC at 1 year: 0.724; at 3 years: 0.841; at 5 years: 0.812; CGGA‐325 cohort, AUC at 1 year: 0.770; at 3 years: 0.865; at 5 years: 0.885; GSE16011, AUC at 1 year: 0.768; at 3 years: 0.868; at 5 years: 0.819; the Rembrandt cohort, AUC at 1 year: 0.705; at 3 years: 0.802; at 5 years: 0.813) (Figure S2A–F). In addition, the risk heatmap suggested that survival time decreased with an increasing risk score (Figure S2G). Based on the average C‐index of the six cohorts, we ultimately selected the best consensus prognostic model for glioma. We also compared the performance of the artificial intelligence‐derived prognostic signature with that of 10 published signatures in predicting patient prognosis.^24^, ^25^, ^26^, ^27^, ^28^, ^29^, ^30^, ^31^, ^32^, ^33^ The cell death‐related risk signature exhibited distinctly superior accuracy to that of the 10 other published signatures (Figure 1N).

### 3.2 The cell death score represents an independent adverse prognostic factor in glioma

**FIGURE 2:**
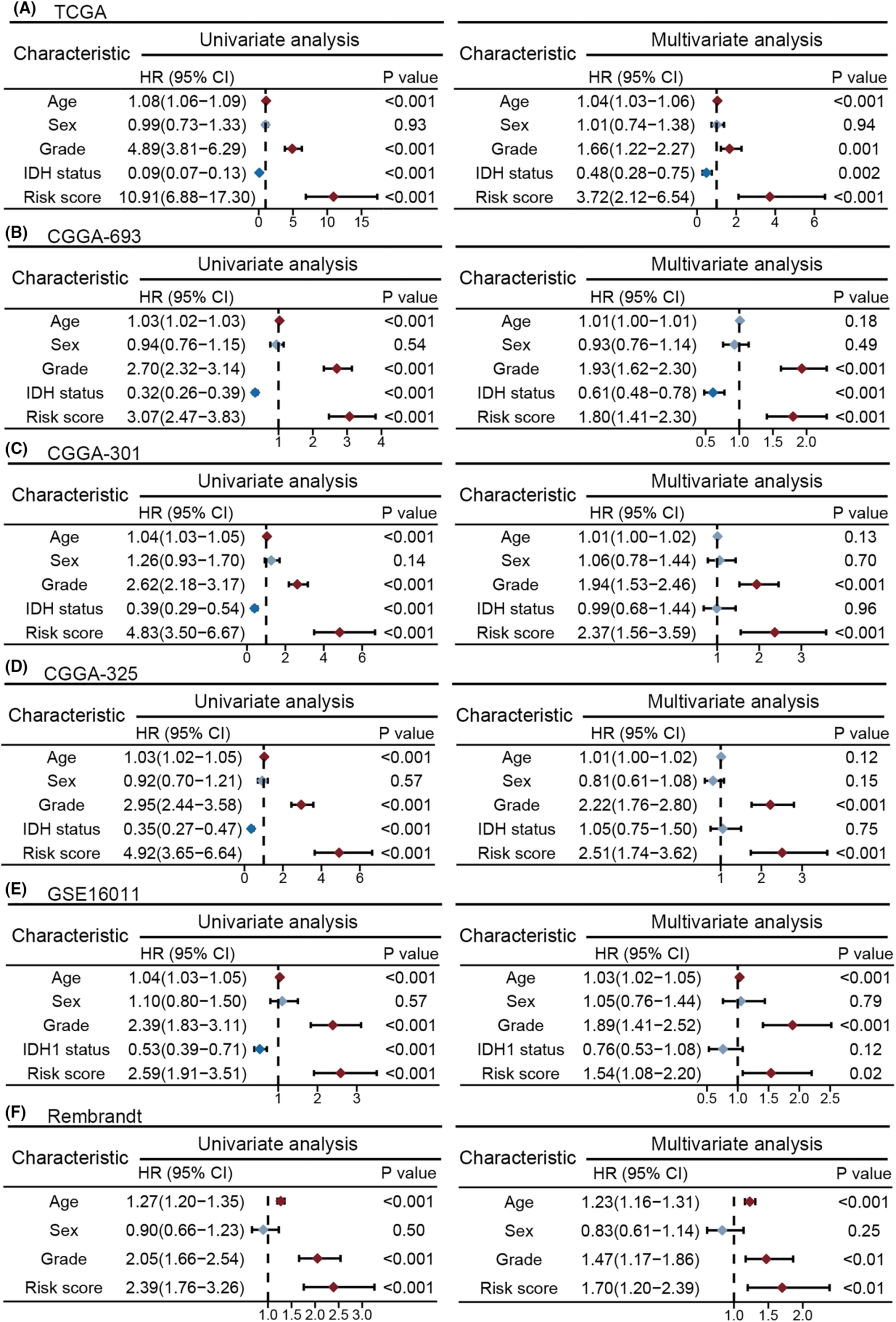
The cell death‐associated score was an independent prognostic factor. (A‐E) Univariate and multivariate Cox regression analyses demonstrated that the risk score was a significant variable for OS, independent of clinicopathological features, in the Cancer Genome Atlas (A), CGGA‐693 (B), CGGA‐301 (C), CGGA‐325 (D), GSE16011 (E) and Rembrandt (F) cohorts. CI, confidence interval; HR, hazard ratio.

Univariate and multivariate Cox regression analyses indicated that the cell death‐related score was a prognostic factor for OS that was independent of clinical variables (age, sex, WHO grade and/or IDH mutation status) in the TCGA cohort (univariate Cox regression analysis: OS, hazard ratio (HR) = 10.91, 95% CI = 6.88–17.30; multivariate Cox regression analysis: OS, HR = 3.72, 95% CI = 2.12–6.54) (*p* < 0.05; Figure 2A). Similar findings were observed in the GSE16011, CCGA‐693, CCGA‐301, CCGA‐325 and Rembrandt cohorts (univariate Cox regression analysis, HR > 2.00; multivariate Cox regression analysis, HR > 1.00) (*p* < 0.05; Figure 2B–F).

### 3.3 Development, performance evaluation and validation of the cell death‐based nomogram for predicting the prognosis of glioma patients

**FIGURE 3:**
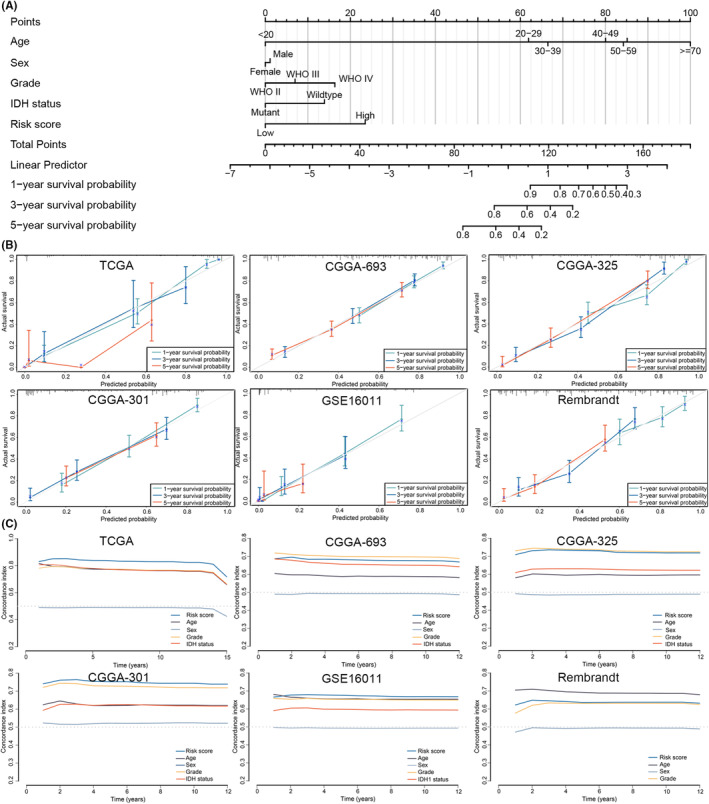
Construction and assessment of a cell death‐associated nomogram (A) The construction of a nomogram to predict 1‐year, 3‐year, and 5‐year OS in the Cancer Genome Atlas (TCGA) cohort; (B) Calibration curve for the nomogram model for 1‐year, 3‐year, and 5‐year OS in the TCGA, CGGA‐693, CGGA‐301, CGGA‐325, GSE16011 and Rembrandt cohorts; (C) Time‐dependent ROC curves comparing the prognostic accuracy of the risk score with that of other clinicopathological risk factors in the six cohorts.

The risk score was integrated with other clinical variables (age, sex, WHO grade and IDH mutation status) to construct a nomogram in the TCGA cohort (Figure 3A). ROC and calibration plots were generated to evaluate the prediction accuracy and discrimination ability of the model, respectively. A calibration curve was used to determine the discriminative ability of the nomogram, which suggested that the nomogram displayed good agreement between the prediction and observation results in the TCGA cohort, which was confirmed in the other five cohorts (Figure 3B). In addition, the ROC curve analysis indicated the superiority of the nomogram for tumour prediction in the six cohorts (Figure 3C).

### 3.4 Analysis of functional enrichment and somatic mutations in different risk groups

**FIGURE 4:**
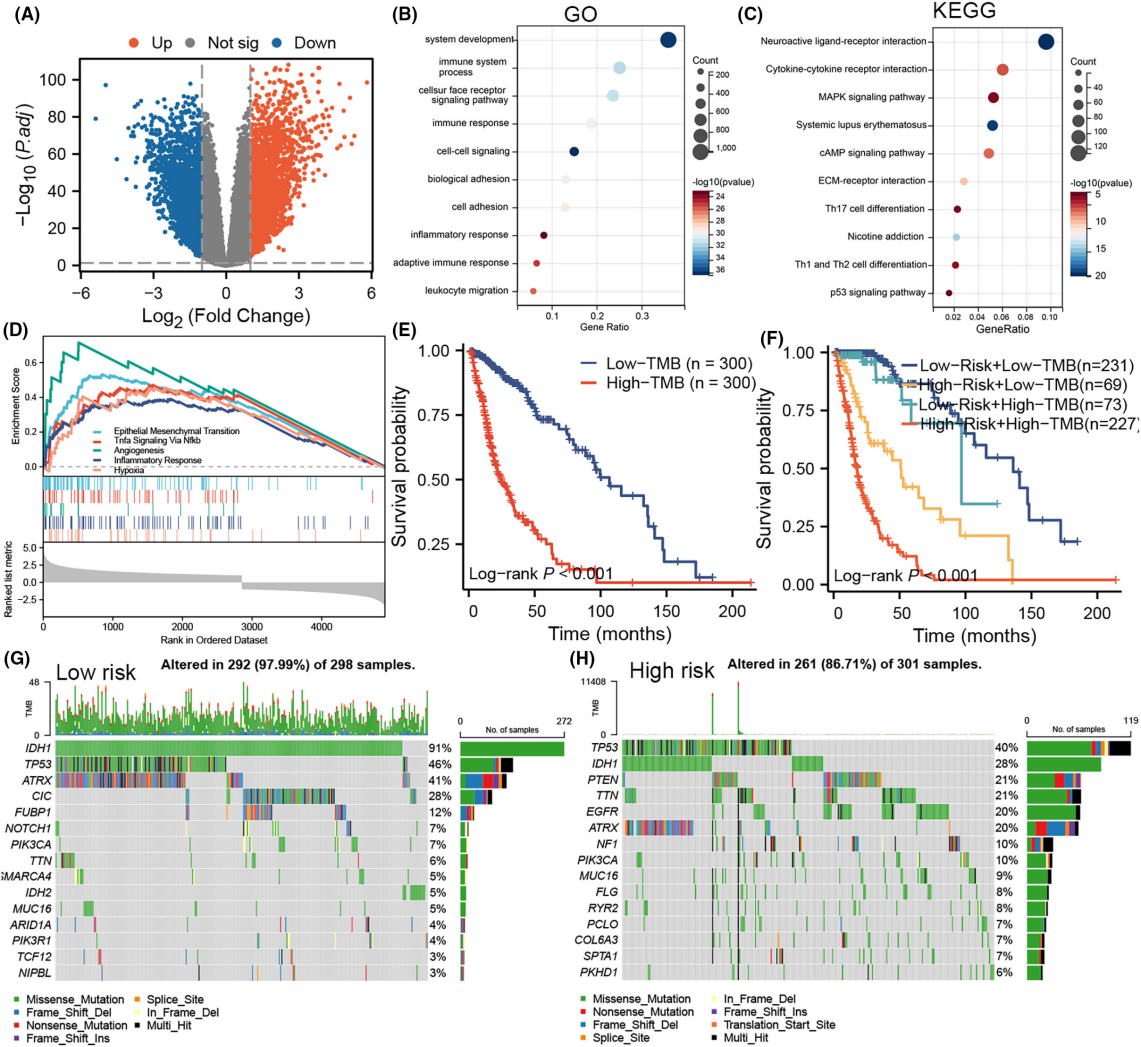
Analysis of DEGs, functional enrichment and somatic mutations in gliomas in different risk groups (A) Volcano plot showing differentially expressed genes between gliomas in different risk groups in the Cancer Genome Atlas cohort; (B, C) Gene Ontology and Kyoto Encyclopaedia of Genes and Genomes enrichment analysis; (D) GSEA. Thresholds with a nominal *p* < 0.05 and an FDR < 25% were used to determine the significance of the enrichment score (ES). (E) Kaplan–Meier survival curves of OS in patients with high and low TMB. (F) Kaplan–Meier survival curves of OS in patients in different risk groups and with different TMB. (G, H) Comparison of somatic mutations in the high‐ and low‐risk groups.

After identifying DEGs between the two groups in the TCGA cohort, 3854 genes were identified as significantly upregulated, and 3205 genes were identified as significantly downregulated (Table S5) (Figure 4A). GO functional and KEGG pathway enrichment analyses indicated that the DEGs were involved in multiple biological pathways, such as the ‘immune system process’, ‘immune response’ and ‘MAPK signalling pathway’ (Figure 4B,C). Subsequently, according to GSEA, the genes in the TCGA cohort were enriched mainly in epithelial‐mesenchymal transition, ‘TNFA signalling via NFKB’, ‘Inflammatory response’, ‘Angiogenesis’ and ‘Hypoxia’ (NES >1, adjusted *p* < 0.05; Figure 4D). A significant difference in prognosis was observed in the TCGA cohort (log‐rank test *p* < 0.0001; Figure 4E), in which patients with gliomas and a low TMB had longer survival than those with high TMB. We also found that patients with gliomas with a low TMB and risk score had significantly longer OS than those with a high TMB and risk score (log‐rank test *p* < 0.001; Figure 4F). We then explored genomic alterations in gliomas in both groups. Patients with gliomas of different risk scores exhibited distinct mutation characteristics. The mutation frequency of IDH1 was significantly higher in low‐risk samples than in high‐risk samples (91% vs. 28%), TP53 (46% vs. 40%) and ATRX (41% vs. 20%) (Figure 4G,H).

### 3.5 Associations between the risk score and immune cells and immune checkpoint molecules

**FIGURE 5:**
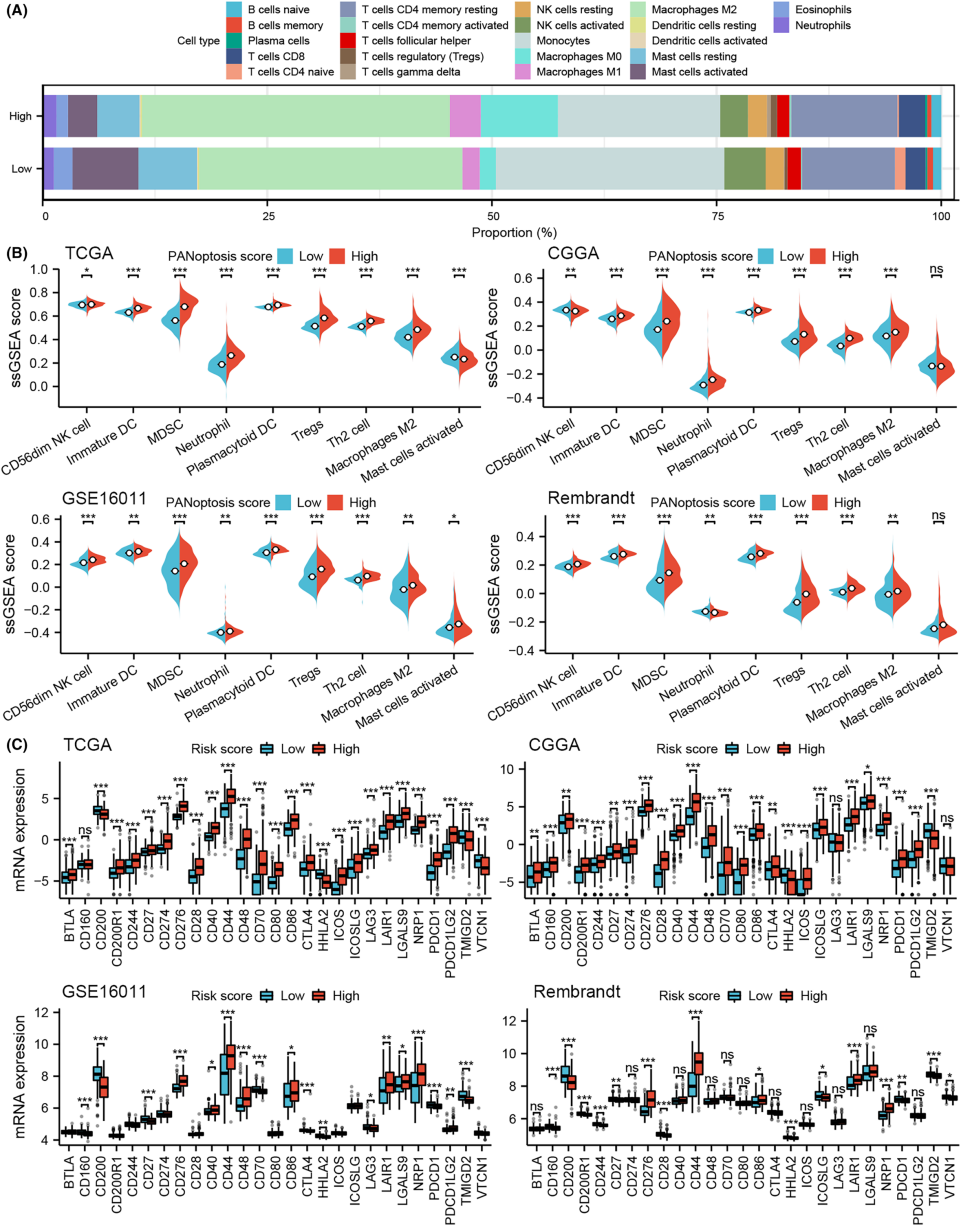
Cellular and molecular differences in gliomas of different cell death‐associated risk groups (A) The abundance of tumour immune infiltration factors in gliomas in the high‐ and low‐risk groups of the Cancer Genome Atlas (TCGA) cohort. (B) Association between risk score and the abundance of protumor immune cells according to the single sample gene set enrichment analysis method in the TCGA, CGGA, GSE16011 and Rembrandt cohorts. (C) Correlation between risk score and the expression levels of immune checkpoint mRNAs in the TCGA, CGGA, GSE16011 and Rembrandt cohorts. Differences between the two groups were compared by Student's *t*‐tests, and the *p* values are labelled above each boxplot with asterisks (ns, no signature; *p < 0.05; **p < 0.01; and ***p < 0.001).

The CIBERSORT algorithm was applied to deconvolute bulk RNA‐seq profiles into 22 cell‐type scores in each glioma sample.^34^ The results showed that M2 macrophages accounted for a higher proportion of gliomas in the high‐risk group than in the low‐risk group, while CD8+ cells accounted for a lower proportion. Similarly, heterogeneity of other immune cells was also detected between gliomas in the two groups (Figure 5A). M2 macrophages are important components of the glioma microenvironment and are closely associated with a poor prognosis.^36^ Because immunosuppressive cells may contribute to the remodelling of the immunosuppressive microenvironment in glioma, correlations between the cell death score and protumor immune cells should be determined. We found that gliomas with high scores, except for those with CD56dim NK cells, exhibited denser immunosuppressive cells in the TCGA cohort (*p* < 0.05; Figure 5B). Similar results were observed in the CGGA, GSE16011 and Rembrandt cohorts (Figure 5B, *p* < 0.05).

Immune checkpoint blockade therapy has achieved promising therapeutic effects on multiple tumours.^37^, ^38^, ^39^ We thus explored the correlation between the cell death score and the expression of 27 immune checkpoint molecules and ligands in gliomas. We found that a large proportion of immune checkpoints were upregulated in the high‐risk group. A similar result was observed in the other cohorts (*p* < 0.05; Figure 5C). However, not all immune checkpoint molecules were consistently expressed in gliomas in the high‐risk cohort. For example, CD160 was highly expressed in the high‐risk group in the GSE16011, CGGA and Rembrandt cohorts (*p* < 0.05; Figure 5C), while there was no significant difference in CD160 expression in the TCGA cohort (*p* > 0.05; Figure 5C).

### 3.6 MYD88 was a hub gene associated with a cell death‐based prognostic model

**FIGURE 6:**
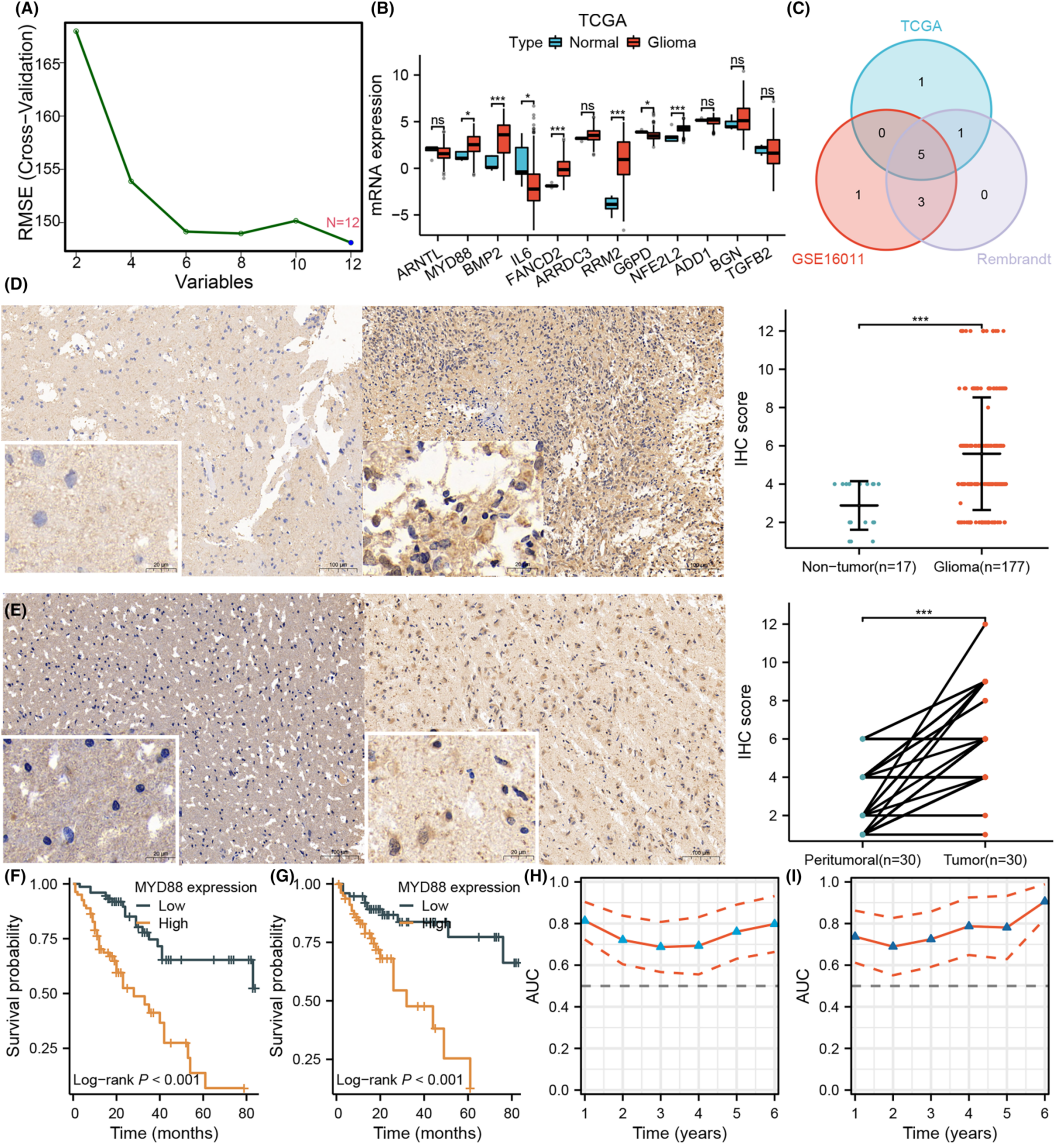
The expression level and prognostic value of MYD88 in glioma. (A) Identification of hub genes by the root mean squared error. (B) Expression levels of 12 cell death‐associated genes in normal tissues and gliomas in the Cancer Genome Atlas (TCGA) cohort. (C) Shared differential genes in the TCGA, GSE16011 and Rembrandt cohorts. (D) Expression level of MYD88 in normal tissues and gliomas in the in‐house cohort determined by immunohistochemistry (IHC). (E) Expression level of MYD88 in gliomas and matching peritumoral tissues analysed by IHC. Differences between the two groups were compared by Student's *t*‐tests, and the *p* values are labelled above each boxplot with asterisks (ns, not significant; *p < 0.05; **p < 0.01; and ***p < 0.001). (F, G) The Kaplan–Meier curves show the correlation between MYD88 expression PFS (F) and OS (G) in the in‐house cohort determined by IHC. (H, I) Time‐dependent ROC analysis at 1 year, 3 years and 5 years depicted the prediction accuracy of the cell death‐associated score in the in‐house cohort. *p* Values were calculated by the log‐rank test, and *p* < 0.05 was considered to indicate statistical significance.

We performed machine learning to screen 12 genes from cell death‐related risk signatures (Figure 6A). We further determined the clinical implications of these differences by comparing the mRNA expression in both glioma and nonglioma tissues. Only five genes, including MYD88, were markedly upregulated in glioma tissues in the GSE16011, TCGA and Rembrandt cohorts (*p* < 0.05; Figure 6B,C). Considering that MYD88 has the highest risk factor, we next explored the expression level of MYD88 in glioma tissues. Similarly, IHC staining showed that the MYD88 expression level was significantly higher in the 177 glioma tissue samples than in the 17 normal tissue samples (*p* < 0.05; Figure 6B). Importantly, compared with that in 30 pairs of nontumor tissues, the MYD88 expression level was also significantly increased in glioma tissue (*p* < 0.05; Figure 6D). A high MYD88 expression level was significantly related to unfavourable clinical outcomes (OS and PFS) in glioma patients in the external cohort (log‐rank test *p* < 0.05; Figure 6F,G). The time‐dependent ROC curve showed that the AUC at different time points was higher than 0.6, which indicated that the prognostic model was highly effective at distinguishing good or poor prognoses in glioma patients (log‐rank test *p* < 0.05; Figure 6H,I). Therefore, these observations indicated that MYD88 was an unfavourable prognostic marker.

### 3.7 Associations between MYD88 expression and the expression of key markers

**FIGURE 7:**
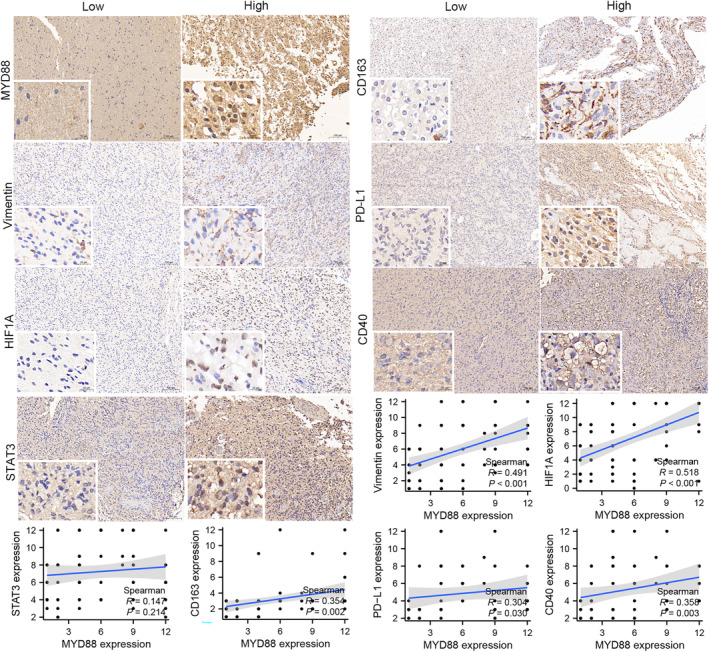
MYD88 expression is correlated with CD163, vimentin, PD‐L1, CD40, STAT3 and HIF1A expression in glioma.

We explored the relationship between MYD88 expression and the expression of multiple key markers, such as vimentin, HIF1A and STAT3. MYD88 was found to be positively related to HIF1A and vimentin expression (vimentin, Spearman *r* = 0.491; HIF1A, Spearman *r* = 0.518) (*p* < 0.05; Figure 7). We did not find a significant correlation between MYD88 and STAT3 expression (*p* > 0.05; Figure 7). M2 macrophage MYD88 expression was positively correlated with CD163 expression (Spearman *r* = 0.354, *p* < 0.05; Figure 7). IHC staining showed that PD‐L1 and CD40 expression increased with increasing MYD88 expression (PD‐L1, Spearman *r* = 0.304; CD40, Spearman *r* = 0.358) (*p* < 0.05; Figure 7).

## 4 DISCUSSION

The glioma microenvironment is generally considered to be highly immunosuppressive and heterogeneous and leads to tumour progression.^40^ However, it is difficult to construct prediction systems that affect the prognosis of glioma patients. The survival time of glioma patients is significantly heterogeneous and depends on tumour grade, and the median survival ranges from 1 to 15 years.^41^ The establishment of a prognostic model is beneficial for predicting patient survival time. In addition, gliomas are also characterized by a tumour microenvironment and molecular heterogeneity.^42^

Immune checkpoint blockade therapy has achieved promising therapeutic effects on multiple tumours.^37^, ^38^, ^39^ However, immunotherapy is effective for only a minority of gliomas, mainly due to resistance to immune‐mediated PCD^10^. Currently, with the increasing appreciation of the importance of PCD in the tumour microenvironment, distinct molecular features of PCD pathways have been recognized. All PCD pathways complement each other and can function together in combination with stimuli in the environment. The importance of various modes of death in glioma patients is worth discussing. In this study, we constructed glioma risk assessment models with genes associated with cell death and found that patients in the high‐risk group had a higher concentration of immunosuppressive cells, such as M2 macrophages, and a smaller proportion of antitumor immune cells, including CD8^+^ cells, in the tumour microenvironment. These results suggest that cell death can form an immunosuppressive microenvironment, which is consistent with previous findings that only a minority of gliomas benefit from immunotherapy due to resistance to immune‐mediated PCD^10^. In addition, we explored the expression of immune checkpoint receptors and ligands in glioma patients in the high‐risk group and low‐risk group. The results showed that a large proportion of immune checkpoint molecules were expressed at higher levels in the high‐risk group than in the low‐risk group. These findings suggest that glioma patients in the high‐risk group may be more sensitive to immune checkpoint inhibitor therapy; thus, immune checkpoint inhibitor therapy may be a promising treatment for glioma patients with high‐risk scores.

To predict the prognosis of glioma patients, a cell death‐related scoring system was constructed in the TCGA training set based on the mRNA expression of 13 cell death‐related genes (Figure 1); its prognostic predictive ability was fully validated in more than 2000 patients from other independent glioma cohorts (CGGA, GSE16011, CGGA‐301, CGGA‐325 and Rembrandt cohorts) (*p* < 0.05; Figure 1). Furthermore, the risk score served as a robust prognostic factor, independent of clinical variables (*p* < 0.05; Figure 2). We constructed a nomogram by integrating the risk score with other clinicopathological factors (Figure 3A), which exhibited good agreement between the nomogram predictions and practical observations according to the calibration plots (Figure 3B). Moreover, the ROC curve indicated the superiority of the nomogram in terms of its ability to predict survival in the six cohorts (Figure 3C). Our results demonstrated the clinical validation and application of risk scores.

Functional enrichment analysis showed that DEGs were enriched in inflammatory processes (Figure 4B–D), which indicated that differences stratified by the cell death‐related signature were involved in the inflammatory process. Moreover, patients with high scores demonstrated denser infiltration of protumor immune cells, such as neutrophils, Tregs and M2 macrophages, which are involved in immunotherapy resistance. We found that most of the immune checkpoint genes were upregulated in the high‐risk group (*p* < 0.05; Figure 5C). A similar result was observed in the other cohorts (*p* < 0.05; Figure 5C). Therefore, our risk classification helps predict the efficacy of immunotherapy for gliomas. However, not all immune checkpoint molecules were consistently expressed in gliomas in the high‐risk cohort. For example, CD160 was highly expressed in the high‐risk group in the GSE16011, CGGA and Rembrandt cohorts (*p* < 0.05; Figure 5C), while CD160 expression was not significantly different in the TCGA dataset (*p* > 0.05; Figure 5C).

To validate the reproducibility of the cell death‐associated genes in the independent cohorts, MYD88 was selected as the research object for in‐depth study via machine learning. MYD88 was highly expressed in glioma (*p* < 0.05; Figure 6A–C), which was substantiated in real‐world samples by IHC (*p* < 0.05; Figure 6D,E). Furthermore, a high MYD88 expression level was significantly related to unfavourable clinical outcomes (overall survival [OS] and progression‐free survival [PFS]) in the in‐house cohort (log‐rank test *p* < 0.05; Figure 6F,G). Based on previous reports, MYD88 is linked to carcinogenesis. The MYD88 knockout inhibited proliferation, enhanced apoptosis and decreased metastasis in tumours.^43^, ^44^, ^45^ MYD88 gain‐of‐function mutations improve tumour cell growth in non‐Hodgkin lymphomas.^46^ Therefore, MYD88 may play a vital role in tumour development. It has been reported that the other four cell death‐related hub genes, including FANCD2, RRM2, BMP2 and NFE2F2, are closely related to tumour immunity and are upregulated in tumour tissues. For example, FANCD2 was upregulated in pancreatic adenocarcinoma and could be an effective biomarker for prognostic recognition, immune efficacy evaluation and mRNA vaccination in pancreatic adenocarcinoma patients.^47^ RRM2 regulates antitumor immune responses, and knockdown of RRM2 enhances the antitumor efficacy of PD‐1 blockade in renal cancer.^48^ BMP2 is highly expressed in glioma and functions as an enhancer of RNA‐regulated immune genes.^49^ In lung squamous cell carcinomas, alterations in KEAP1‐NFE2L2 could affect antitumor immune responses.^50^ These reports are consistent with our results, indicating that the cell death‐related hub genes that were identified by machine learning can be used to assess the prognosis and immune infiltration landscape of glioma patients well.

MYD88 can regulate innate immunity by acting directly on Toll‐like receptors (TLRs) and cytokine receptors, NF‐κB signalling and MAPK signalling.^51^ TLRs are a superfamily of pattern recognition receptors that activate innate and adaptive immunity.^52^ All TLRs, except TLR3, independently regulate the immune response via the MYD88‐mediated pathway, while TLR3 activates the MYD88‐independent pathway to produce inflammatory cytokine products.^53^ In addition, MYD88 might promote tumour cell survival via NF‐κB signalling in colorectal carcinoma cells.^54^ TLRs can restore tumour differentiation in glioma cells and transform microglia into a glioma‐supportive phenotype.^55^, ^56^ These findings show that MYD88 plays an important role in glioma. MYD88 expression is reported to be significantly related to OS, and patients with higher MYD88 expression have more M2‐type macrophage infiltration.^57^ In addition, we used IHC to analyse MYD88 expression and the expression level of CD163, which is a surface marker of M2 macrophages. M2 macrophages are important components of the glioma microenvironment and are closely related to the clinical outcome of patients.^36^ M2 macrophages interact with glioma cells, which contributes to the rapid progression of gliomas.^58^ We found via IHC that MYD88 expression is correlated with CD163, vimentin, PD‐1/PD‐L1, CD40, STAT3 and HIF1A expression in glioma (Figure 7). These findings confirm that MYD88 may be involved in regulating the complex tumour microenvironment.

Although our scoring system showed excellent performance for glioma prognosis in both training and validation cohorts, there are still some limitations and shortcomings to be solved. First, this scoring system needs to be validated by experimental data; second, the exact mechanism underlying PANoptosis need to be explored; Finally, more functional experiments are needed to validate the role of MYD88 in the glioma microenvironment. In addition, increasing evidence has shown that miRNAs may affect gene expression and disease progression, and Some research reports on the construction of disease computational models by miRNAs, such as an updated review of advances in microRNAs and complex diseases: towards systematic evaluation of computational models,^59^, ^60^, ^61^, ^62^ indicate the direction for our next research work. In future research, we plan to use miRNAs, which target 12 genes for cell death‐related risk signatures, to explore the accuracy of this model in evaluating glioma prognosis and the effectiveness of immune molecular targets.

## 5 CONCLUSION

We developed a cell death‐related risk score that reflects distinct clinical, molecular and immune characteristics and provided a precise stratification of prognosis and immunotherapy efficacy in glioma patients. MYD88 was found to be an outstanding representative that might play an important role in glioma.

## AUTHOR CONTRIBUTIONS

**Quanwei Zhou:** Data curation (lead); writing – original draft (lead); writing – review and editing (lead). **Fei Wu:** Writing – review and editing (equal). **Wenlong Zhang:** Formal analysis (equal); methodology (equal). **Youwei Guo:** Investigation (equal); visualization (equal). **Xingjun Jiang:** Funding acquisition (equal); project administration (equal). **Xuejun Yan:** Formal analysis (equal); investigation (equal); project administration (equal); supervision (equal). **Yiquan Ke:** Funding acquisition (equal); project administration (equal).

## FUNDING INFORMATION

This study was supported by the National Natural Science Foundation of China Grant Number 82072762 (KY); Grant Number 81472355 (XJ), the China Postdoctoral Science Foundation funded project Grant Number 2023M741587 and Natural Science Foundation of Hunan Province Grant Number 2024JJ6262. We are sincerely grateful to those who created and maintained these public databases.

## CONFLICT OF INTEREST STATEMENT

The authors confirm that there are no conflicts of interest.

## Supporting information


Figure S1.

Figure S2.

Figure S3.

Table S1.

Table S2.

Table S3.

Table S4.

Table S5.

## Data Availability

Publicly available datasets were analysed in this study. The datasets include CGGA, TCGA, GSE16011 and Rembrandt databases.
