# Supplementary figures and images for: Machine learning‐based identification of a cell death‐related signature associated with prognosis and immune infiltration in glioma
Source: J Cell Mol Med. 2024 Jun 7;28(11):e18463. doi: 10.1111/jcmm.18463 (PMC11157676; doi:10.1111/jcmm.18463)

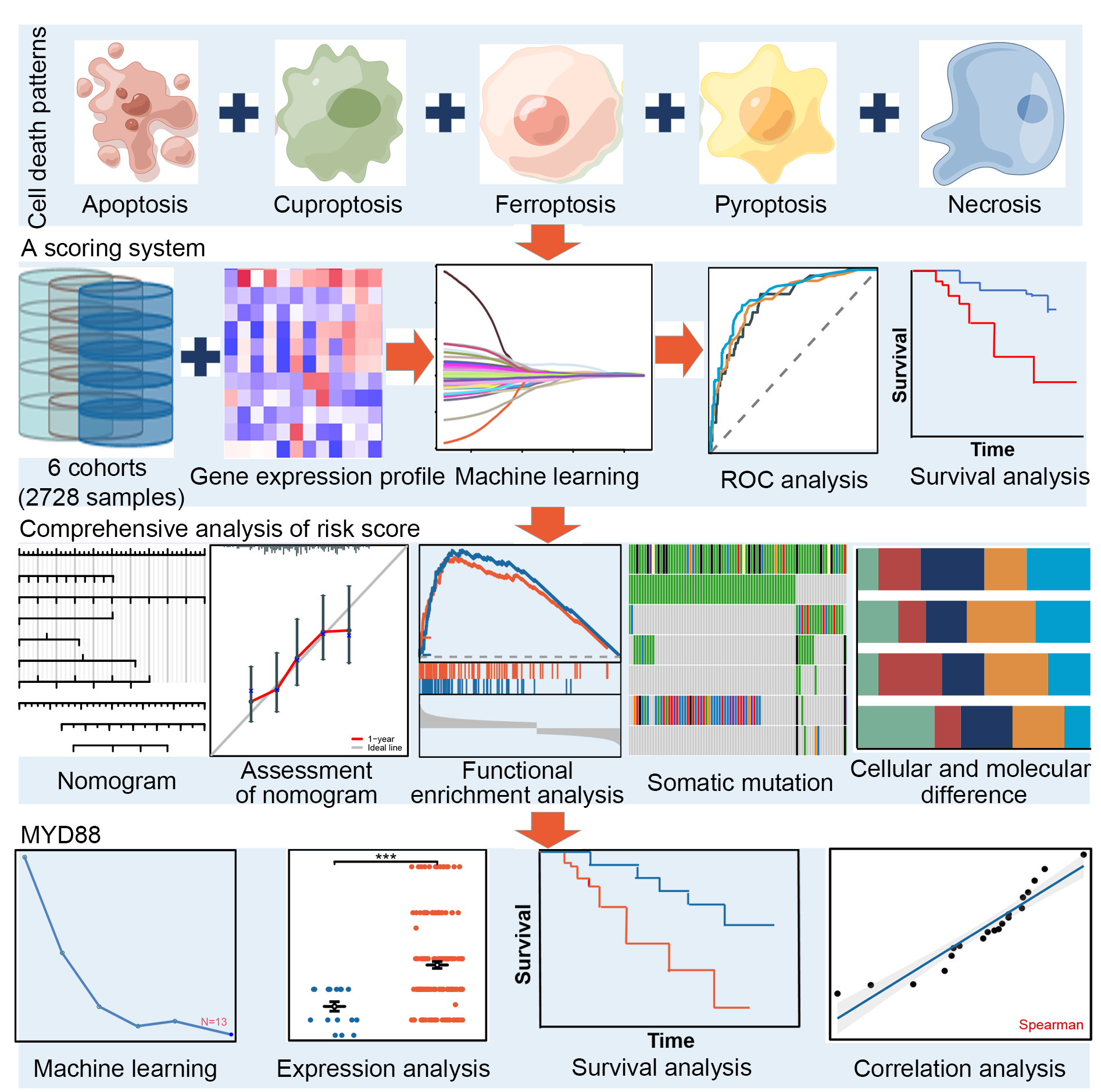

Supplement: Supplementary file 1 — Figure S1. [file JCMM-28-e18463-s002.tif]

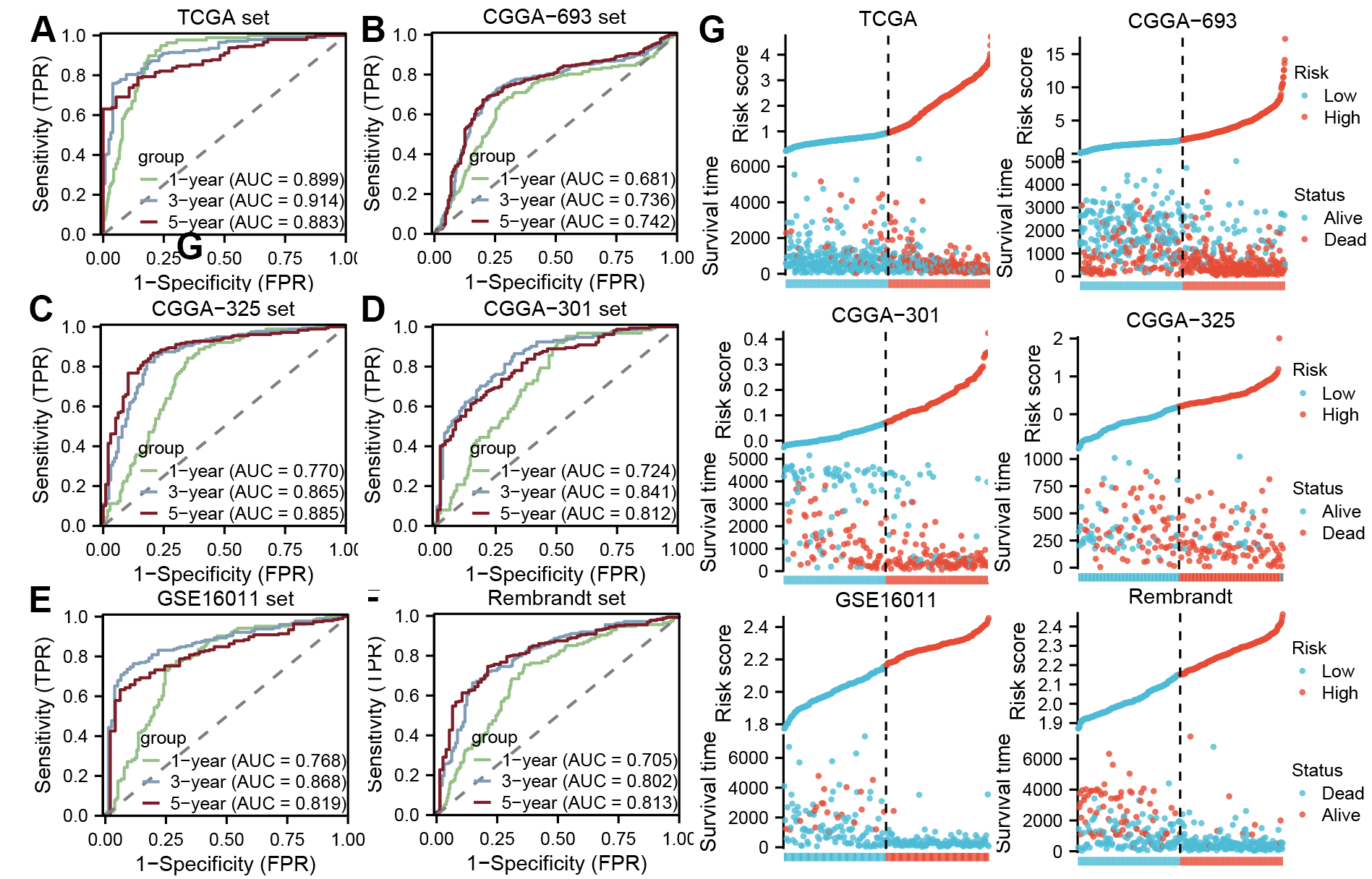

Supplement: Supplementary file 2 — Figure S2. [file JCMM-28-e18463-s006.tif]

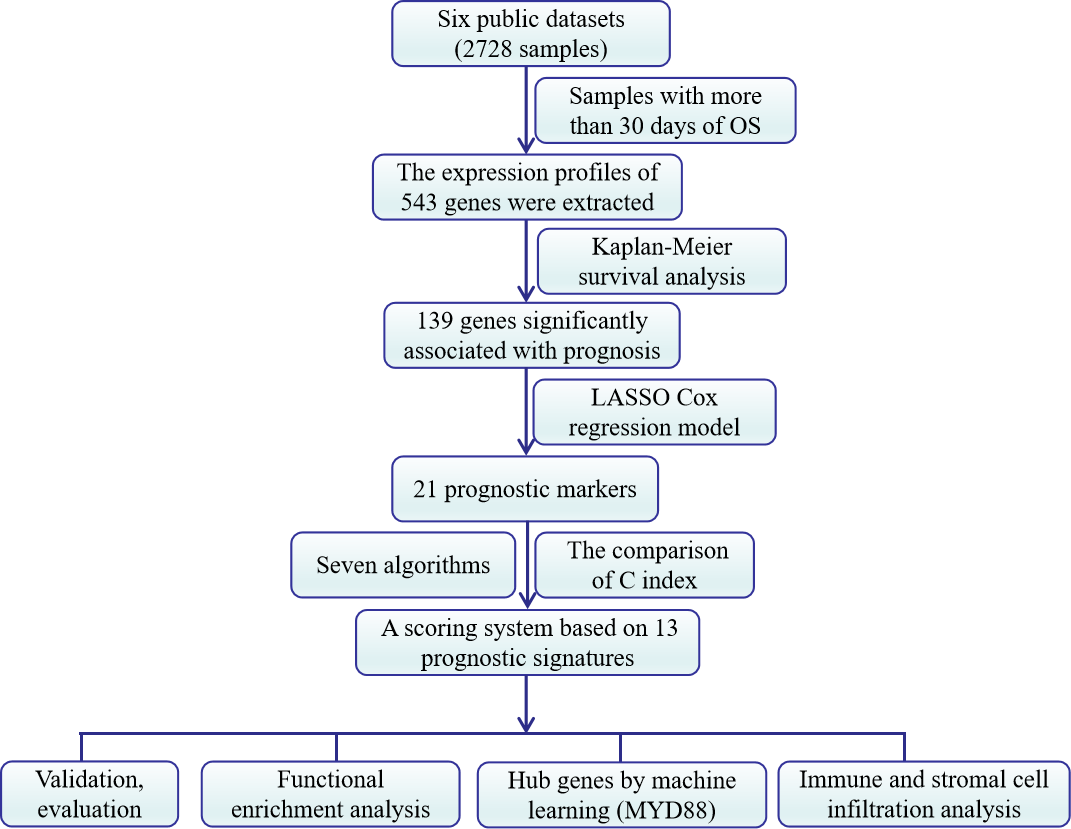

Supplement: Supplementary file 3 — Figure S3. [file JCMM-28-e18463-s008.tif]
